# Supplementary material for: Anxiety and depression among people living in quarantine centers during COVID-19 pandemic: A mixed method study from western Nepal
Source: PLoS One. 2021 Jul 9;16(7):e0254126. doi: 10.1371/journal.pone.0254126 (PMC8270129; doi:10.1371/journal.pone.0254126)
Supplement: S3 Appendix — (DOCX) [file pone.0254126.s004.docx]

**Questionnaire In English**

**Part A**

**District M Rural/Municipality M Ward No M**

**Village/ Tole M**

**Name of Interviewer: Date of Interview (DD/MM/YY):**

**Time of Interview:**

Part A: **Socio-demographic information**

| **Q.N** | **Question** | **Response** |
| --- | --- | --- |
| !)! | Family Members in your family? |  |
| !)@ | What is your caste/ ethnicity? Please choose from the given list | \| Bramin/Chettri/ Thakuri \| 1 \| \| --- \| --- \| \| Madhesi \| 2 \| \| Dalit Hill \| 3 \| \| Dalit Terai \| 4 \| \| Janjati Hill \| 5 \| \| Janjati Terai \| 6 \| \| Muslim \| 7 \| \| Others……………. \| 8 \| |
| !)# | What is your religion? | \| Hindu \| 1 \| \| --- \| --- \| \| Buddhist \| 2 \| \| Muslim \| 3 \| \| Kirat \| 4 \| \| Cristain \| 5 \| \| Others………………… \|  \| |
| !)$ | What is your current age? (Write your completed years) | Age In years: |
| !)% | What is the grade that you have passed? | Completed Grade================   \| Illilterate \| 1 \| \| --- \| --- \| \| Formal education \| 2 \| |
| !)^ | How many family members are dependent on your salary? | ===========================================  -Write in Numbers_ |
| !)& | Is your family nuclear or joint? | \| Nuclear \| 1 \| \| --- \| --- \| \| **Joint** \| 2 \| |
| !)* | Do you have any other source of income rather than household works? | \| Yes \| 1 \| \| --- \| --- \| \| No \| 2 Go to 111 \| |
| !)( | What is your current employment status? | ==============================================   - Please specify \_ |
| !!) | What is your current monthly income | ================================== - Write in NPR_  If don’t wish to answer write 99 |
| !!! | Do you have children? | \| Yes \| 1 \| \| --- \| --- \| \| No \| 2 Go to 113 \| |
| !!# | What was the family monthly income of your for last years? (In Nepali Rupees) (Family monthly income denotes the total of all income of those residing under one kitchen) | Write in NPR…………………………… |
| !!$ | What are the following accessories you have in your home? | \|  \| Yes \| No \| \| --- \| --- \| --- \| \| Electricity \| 1 \| 2 \| \| Radio \| 1 \| 2 \| \| Tv \| 1 \| 2 \| \| Landline Phone \| 1 \| 2 \| \| Mobile Phone \| 1 \| 2 \| \| Computer \| 1 \| 2 \| \| Internet \| 1 \| 2 \| \| Freeze \| 1 \| 2 \| \| Cable TV \| 1 \| 2 \| |
| !!% | What is the frequency that you read newspaper? | \| At least once a week \| 1 \| \| --- \| --- \| \| Less than once a week \| 2 \| \| Never \| 3 \| |
| !!^ | s] tkfO{Fn] /]l8of] sDtLdf xKtfsf] Psk6s, xKtfdf Psk6seGbf sd ;'Gg'x'G5< jf ;'Gg' g} ;'Gg' x'Fb}g < | \| At least once a week \| 1 \| \| --- \| --- \| \| Less than once a week \| 2 \| \| Never \| 3 \| |
| !!& | s] tkfO{Fn] 6]lnlehg sDtLdf xKtfsf] Psk6s, xKtfdf Psk6seGbf sd ;'Gg'x'G5 < jf ;'Gg' g} ;'Gg' x'Fb}g < | \| At least once a week \| 1 \| \| --- \| --- \| \| Less than once a week \| 2 \| \| Never \| 3 \| |
| !!* | Do you have account in social media? | \| Yes \| 1 \| \| --- \| --- \| \| No \| 2 Go to QN 120 \| |
| !!( | What is the frequency that you use social media? | \| At least once a week \| 1 \| \| --- \| --- \| \| At least once in a day \| 2 \| \| At least once in 15 days \| 3 \| \| At least once in a month \| 4 \| \| I have account but don’t use \| 5 \| |
| !@) | Do you have any account in cooperative? | \| Yes \| 1 \| \| --- \| --- \| \| No \| 2 \| |
| !@! | Do you have any property in your name? | \| Home \| 1 \| \| --- \| --- \| \| Land \| 2 \| \| Others (Please specify) \| 3 \| \| Nothing I have \| 4 \| |
| !@@ | Who decides on spending your monthly income in your house? | \| Yourself \| 1 \| \| --- \| --- \| \| Your wife/Husband \| 2 \| \| Both together \| 3 \| \| Father and Mother \| 4 \| \| I don’t earn any money \| 5 \| |

**Part B**

Now, I am going to discuss about the symptoms of an individual having Depression. Please tell me how much have you been suffering from those symptoms during past 2 weeks?

**BDI. 1** In the past two weeks, how often were you become sad?

Never………….0

A little….1

Always……..2

Could not stand…..3

**BDI. 2** In the past two weeks, how often were you disappointed? (For instance, expected thing about the future did not happen)

Never………….0

A little…….….1

Always……..2

Could not stand …..3

**BDI.3** In the past two weeks, how often did you feel like a failure about your life?

Never………….0

A little…….….1

Always……..2

Could not stand/ completely felt like a failure …..3

**BDI. 4** In the past two weeks, how often were you unsatisfied with life?

Never………….0

A little………….1

Almost all time……..2

Became unsatisfied in every little thing of life…..3

**BDI. 5** In the past two weeks, how often did you have guilt feelings?

Never………….0

A little….1

Almost all time ……..2

Had guilt feelings in every little thing of life …..3

**BDI. 6** In the past two weeks, how often did you feel you should have been punished? (When self cannot do anything and when things get destroyed)

Never………….0

A little….1

Almost all time ……..2

In every little thing of life …..3

**BDI. 7** In the past two weeks, how often did you feel upset?

Never………….0

A little….1

Always……..2

Hatred myself…..3

**BDI. 8** In the past two weeks, how often did you blame yourself about the past mistakes?

Never………….0

A little….1

Almost all time……..2

Always....3

**BDI. 9** In the past two weeks, how often did you try to hurt yourself? (For example, want to do suicide, want to take drugs)

Never tried to hurt myself………….0

Wanted to hurt myself, but could not….1

Wanted to kill myself……..2

Would kill myself if I had the chance…..3

**BDI. 10** In the past two weeks, how often did you cry?

Did not cry at all………….0

Sometimes cried….1

Cry over every little things...2

Felt like crying, but I could not…..3

**BDI. 11** In the past two weeks, how often did you feel irritating?

Never felt irritating………….0

Felt more irritating than usual….1

Always felt irritating……..2

Could not stand…..3

**BDI. 12** In the past two weeks, how often did you lost your interest? (For example, enjoy in playing with other, doing work)

Had never lost interest………….0

Had less interest…..….1

Had lost interest most of the time……..2

Always lost interest…..3

**BDI. 13** In the past two weeks, how often did you feel difficulty in taking decision?

Never felt difficulty………….0

Little bit difficulty……….1

Felt much difficulty……..2

Always felt difficulty…..3

**BDI. 14** In the past two weeks, how often did you feel worthless?

Never felt worthless………….0

Felt little bit worthless….1

Felt more worthless……..2

Always felt worthless…..3

**BDI.15** In the past two weeks, how often did you work?

Worked as usual………….0

Could not work as usual….1

Had difficulty in working……..2

Could not do any work at all…..3

**BDI. 16** In the past two weeks, how often did you sleep?

Slept as usual………….0

Could not sleep as usual….1

Woke up time to time and could not get back to sleep……..2

Could not sleep at all…..3

**BDI.17** In the past two weeks, how often did you get tired?

Never got tired………….0

Got more tired than usual ….1

Got tired whatever I did……..2

Could not do anything because of tiredness …..3

**BDI.18** In the past two weeks, how often had you lost your appetite?

Appetite same as usual………….0

Appetite somewhat less than usual….1

Appetite much less than usual……..2

Had no appetite…..3

**BDI. 19** In the past two weeks, how often did you feel about your weight loss?

No weight loss………….0

Had lost weight (5 K.g) ….1

Had lost weight (10 K.g)……..2

Drastic weight loss (15 K.g)…..3

**BDI. 20** In the past two weeks, how often were you worried about your health?

Was not worried at all………….0

Was worried sometimes….1

Was worried much of time and could not think of anything……..2

Was always worried, so unconscious about it…..3

**BDI. 21** In the past two weeks, how often did you think of your partner?

Did not think about him/her like before………….0

Did not think less than before….1

Did not care much……..2

Did not care at all…..3

**Part C**

Now, I am going to discuss about the symptoms of an individual having Anxiety. Please tell me how much have you been suffering from those symptoms during past 2 weeks?

**BAI.1** In the past two weeks, how often did your body tingle?

Not at all………….0

Mildly…...1

Moderately, but I could stand it……..2

Severely, I could not stand it…..3

**BAI.2** In the past two weeks, how often did your body feel hot?

Not at all………….0

Mildly….1

Moderately, but I could stand it……..2

Severely, I could not stand it…..3

**BAI.3** In the past two weeks, how often did your legs wobble?

Not at all………….0

Mildly….1

Moderately, but I could stand it……..2

Severely, I could not stand it…..3

**BAI.4** In the past two weeks, how often did you face difficulty to relax?

Not at all………….0

Mildly….1

Moderately, but I could stand it……..2

Severely, I could not stand it…..3

**BAI.5** In the past two weeks, how often did you feel fear of the worst happening?

Not at all………….0

Mildly….1

Moderately, but I could stand it……..2

Severely, I could not stand it…..3

**BAI.6** In the past two weeks, how often did you feel dizzy?

Not at all………….0

Mildly….1

Moderately, but I could stand it……..2

Severely, I could not stand it…..3

**BAI.7** In the past two weeks, how often did your heart pound?

Not at all………….0

Mildly….1

Moderately, but I could stand it……..2

Severely, I could not stand it…..3

**BAI.8** In the past two weeks, how often did you feel restless/ unsteady?

Not at all………….0

Mildly….1

Moderately, but I could stand it……..2

Severely, I could not stand it…..3

**BAI.9** In the past two weeks, how often did you feel terrified?

Not at all………….0

Mildly….1

Moderately, but I could stand it……..2

Severely, I could not stand it…..3

**BAI.10** During past 2 weeks, how much did you feel nervous without any cause?

Not at all………….0

Mildly….1

Moderately, but I could stand it……..2

Severely, I could not stand it…..3

**BAI.11** In the past two weeks, how often did you have feelings of chocking?

Not at all………….0

Mildly….1

Moderately, but I could stand it……..2

Severely, I could not stand it…..3

**BAI.12** In the past two weeks, how often did your hand tremble/crooked?

Not at all………….0

Mildly….1

Moderately, but I could stand it……..2

Severely, I could not stand it…..3

**BAI.13** In the past two weeks, how often did your body feel shaky?

Not at all………….0

Mildly….1

Moderately, but I could stand it……..2

Severely, I could not stand it…..3

**BAI.14** In the past two weeks, how often did you feel fear of losing control?

Not at all………….0

Mildly….1

Moderately, but I could stand it……..2

Severely, I could not stand it…..3

**BAI.15** In the past two weeks, how often did you have difficulty in breathing?

Not at all………….0

Mildly….1

Moderately, but I could stand it……..2

Severely, I could not stand it…..3

**BAI.16** In the past two weeks, how often did you feel fear of dying?

Not at all………….0

Mildly….1

Moderately, but I could stand it……..2

Severely, I could not stand it…..3

**BAI.17** In the past two weeks, how often did you feel scared?

Not at all………….0

Mildly….1

Moderately, but I could stand it……..2

Severely, I could not stand it…..3

**BAI.18** In the past two weeks, how often did you feel the burn in stomach?

Not at all………….0

Mildly….1

Moderately, but I could stand it……..2

Severely, I could not stand it…..3

**BAI.19** In the past two weeks, how often did you faint?

Not at all………….0

Mildly….1

Moderately, but I could stand it……..2

Severely, I could not stand it…..3

**BAI.20** In the past two weeks, how often did your face turned red when you got angry or tensed?

Not at all………….0

Mildly….1

Moderately, but I could stand it……..2

Severely, I could not stand it…..3

**BAI.21** In the past two weeks, how often did you sweat though no heat?

Not at all………….0

Mildly….1

Moderately, but I could stand it……..2

Severely, I could not stand it…..3

**lhNnf sf]8 M gu/kflnsf÷ufpFkflnsf sf]8 M j8f M**

**ufpF÷6f]n M**

**cGt/jftf{ lng]sf] gfd M cGt/jftf{ lnOPsf] ldlt -lbg÷dlxgf÷jif{_M**

**cGt/jftf{ ;'? ePsf] ;do :**

**v08 !M ;fdflhs tyf hg;f+lVos ljj/0f**

| **k\|=g+=** | **k\|Zg** | **k\|ltlqmof** |
| --- | --- | --- |
| !)! | tkfO{sf] 3/df slthgf ;b:ox? x'g'x'G5 <  *-Pp6} efG5fdf vfgf vfg] k\|foM a;f]af; ug]{ ;a} ;b:ox?nfO{ lng'kb{5 ._* |  |
| !)@ | tkfO{sf] hft s] xf] <  *-s[kof tkfO{nfO{ lbO{Psf] ;'rLaf6 hft klxrfg u/L ;xL 7fpFdf uf]nf] nufpg'xf];\_* | \| a\|fXd0f÷If]qL÷7s'/L \| ! \| \| --- \| --- \| \| dw]zL \| @ \| \| blnt kxf8 \| # \| \| blnt t/fO{ \| $ \| \| hghflt kxf8 \| % \| \| hghflt t/fO{ \| ^ \| \| d'l:nd \| & \| \| cGo======================= \| * \| |
| !)# | tkfO{ s'g wd{ dfGg'x'G5< | \| lxGb' \| ! \| \| --- \| --- \| \| af}¢ \| @ \| \| d'l:nd \| # \| \| ls/ft \| $ \| \| lqmlZrog \| % \| \| cGo====================== \| ^ \| |
| !)$ | tkfO{+ slt aif{ x'g'eof] <  -k'/f ePsf] pd]/ v'nfpgxf]; _ | pd]/ M |
| !)% | tkfO{n] slt sIff kf; ug'{ePsf] 5 < | k'/f u/]sf] lzIff================   \| lg/If/ \| ! \| \| --- \| --- \| \| cgf}krfl/s lzIff \| @ \| |
| !)^ | tkfO{sf] cfDbfgLdf kl/jf/sf slthgf ;b:ox? lge{/ x'g'x'G5 < | ===========================================  -;+Vof v'nfpg'xf];\ _ |
| !)& | s] tkfO{+sf] kl/jf/, ;+o'Qm ls Psn s] 5 < | \| Psn a:5' \| ! \| \| --- \| --- \| \| ;+o'Qm?df a:5' \| @ \| |
| !)* | 3/fof;L sfd afx]s s] tkfO{+sf] cGo /f]huf/L 5 < -h:t} :j/f]huf/L, hflu/ cflb_ | \| 5 \| ! \| \| --- \| --- \| \| 5}g \| @ !!! gDa/sf] k\|Zgdf hfg'xf];\ . \| |
| !)( | /f]huf/Lsf] cj:yf s] 5 < | ==============================================  -v'nfpg'xf];\_ |
| !!) | tkfO{{sf] 5'§} /f]huf/Laf6 dfl;s cfDbfgL slt x'G5 < | ================================== -g]?df_  eGg grfx]sf] (( |
| !!! | tkfO{+sf] 5f]/f5f]/Lx? 5g\ < | \| 5 \| ! \| \| --- \| --- \| \| 5}g \| @ !!# gDa/sf] k\|Zgdf hfg'xf];\ . \| |
| !!# | utjif{ tkfO{+sf] kl/jf/sf] cf}ift **dfl;s** cfDbfgL slt lyof]] < -g]kfnL ?k}ofdf_ *-kl/jf/sf] cf};t dfl;s cfDbfgL eGgfn] Pp6} efG;f k\|of]u ug]{ kl/jf/sf ;a} ;b:ox?n] ug]{ cfDbfgLnfO a'em\g'k5{ ._* | g]kfnL ?k}Fof==================== |
| !!$ | s] tkfOsf] 3/df lgDg s'/fx? pknAw 5g\<  ljB't <  /]l8of] <  6]lnlehg<  NofG8nfOg kmf]g<  Dff]afOn kmf]g<  sDKo'6/<  OG6/g]6<  lk\|mh<  s]an 6LeL  *-Ps eGbf a9L pTt/ cfpg ;Sg]_* | \|  \| 5g\ \| 5}gg\ \| \| --- \| --- \| --- \| \| ljB't \| ! \| @ \| \| /]l8of] \| ! \| @ \| \| 6]lnlehg \| ! \| @ \| \| NofG8nfOg kmf]g \| ! \| @ \| \| Dff]afOn kmf]g \| ! \| @ \| \| sDKo'6/ \| ! \| @ \| \| OG6/g]6 \| ! \| @ \| \| lk\|mh \| ! \| @ \| \| s]an 6LeL \| ! \| @ \| |
| !!% | s] tkfO{Fn] kqklqsfx? slQsf] k9\g'x'G5 < | \| sDtLdf xKtfsf] Psk6s \| ! \| \| --- \| --- \| \| xKtfdf Psk6seGbf sd \| @ \| \| slxNo} klg k9\lbg \| # \| |
| !!^ | s] tkfO{Fn] /]l8of] sDtLdf xKtfsf] Psk6s, xKtfdf Psk6seGbf sd ;'Gg'x'G5< jf ;'Gg' g} ;'Gg' x'Fb}g < | \| sDtLdf xKtfsf] Psk6s \| ! \| \| --- \| --- \| \| xKtfdf Psk6seGbf sd \| @ \| \| ;'lGbg \| # \| |
| !!& | s] tkfO{Fn] 6]lnlehg sDtLdf xKtfsf] Psk6s, xKtfdf Psk6seGbf sd ;'Gg'x'G5 < jf ;'Gg' g} ;'Gg' x'Fb}g < | \| sDtLdf xKtfsf] Psk6s \| ! \| \| --- \| --- \| \| xKtfdf Psk6seGbf sd \| @ \| \| x]lb{g \| # \| |
| !!* | s] tkfO{sf] ;f];n ldl8of csfpG6 5< h:tf] ls km];a's, Odf]< | \| 5 \| ! \| \| --- \| --- \| \| 5}g \| @ !@) gDa/sf] k\|Zgdf hfg'xf];\ . \| |
| !!( | tkfO{ ;f];n ldl8ofsf] k\|of]u s'g dfqfdf ug'{x'G5< | \| slDtdf lbgsf] Psk6s \| ! \| \| --- \| --- \| \| slDtdf xKtfsf] Psk6s \| @ \| \| slDtdf !% lbgsf] Psk6s \| # \| \| slDtdf dlxgfsf] Psk6s \| $ \| \| csfpG6 t 5 t/ rnfpFlbg \| % \| |
| !@) | s] s'g} a}+s jf ;xsf/Ldf tkfO{sf] vftf 5< | \| 5 \| ! \| \| --- \| --- \| \| 5}g \| @ \| |
| !@! | s] s'g} ;DklQ tkfO{sf] gfddf 5 < | \| 3/ \| ! \| \| --- \| --- \| \| hUuf \| @ \| \| cGo eP pNn]v ug'{xf];\ ============ \| # \| \| s'g} klg 5}g \| $ \| |
| !@@ | tkfO{n] cfh{g ug{'ePsf] k};f s;/L vr{ ug]{ eGg]af/] s;n] lg0f{o u5{< | \| tkfO{n] \| ! \| \| --- \| --- \| \| tkfO{sf] >LdftLn] \| @ \| \| b'j}hgfn] ;+o'St?kdf \| # \| \| jf cfdfn] \| $ \| \| d}n] k};f cfh{g ulb{g \| % \| |

**v08 @**

**BDI. 1** uPsf] b'O{ xKtfdf tkfO{ slQsf] pbf; - b'MvL_ x'g'x'GYof] <

-lbSsbf/L, lj/Qm, b'MvL_

slxNo} eO{g================================================================================ )

clncln ePF==================================================================================== !

;w}+ ePF ===================================================================================== @

c;x\o ePsf] lyof] ======================================================================= #

**BDI. 2** uPsf] b'O{ xKtfdf tkfO{ slQsf] lg/fz x'g' eof] < -h:t} cfkm'n] ;f]r]sf] h:tf] gx'+bf_ eljiosf] af/]df ;Dem]/_

slxNo} lg/f; eO{g ========================================================================== )

clncln lg/f; ePF============================================================================ !

;w}+ lg/f; ePF ================================================================================ @

c;x\o ePsf] lyof] ======================================================================== #

**BDI.3** uPsf] b'O{ xKtfdf tkfO{nfO{ cfkm\gf] hLjgel/sf] s'/f ;Dem]/ slQsf] c;kmn ePh:tf] dxz'; eof]<

slxNo} dxz'; ePg========================================================================== )

clncln dxz'; eof]========================================================================= !

;w}+ dxz'; eof]======================================================= ======================== @

c;x\o eof]/k"0f{?kn] c;kmn eP h:tf] nfUof] =================================== #

**BDI. 4** uPsf] b'O{ xKtfdf tkfO{ cfkm\gf] hLjg b]lv slQsf] c;Gt'i6 -c;Gtf]if÷lrQ ga'em\g]_ x'g'eof] <

slxNo} c;Gt'i6 eO{g================================================================= )

clncln c;Gtf]if ePF======================================================================== !

;w}+ h;f] c;Gt'i6 ePF =================================================================== @

hLjgsf] x/]s s'/fdf c;Gt'i6 ePF === ============================================== #

**BDI. 5** uPsf] b'O{ xKtfdf tkfO{n] cfkm\gf] lhGbuLsf] nflu cfkm'nfO{ slQsf] bf]lif 7fGg'eof] <

slxNo} bf]lif 7flgg======================================================================= )

clncln bf]lif 7fg]+ ================================================= ========================= !

;w}+ h;f] bf]lif 7fg]+ ===================================================================== @

hLjgsf] x/]s s'/fdf bf]lif 7fg]+ ===================================================== #

**BDI. 6** uPsf] b'O{ xKtfdf tkfO{n] cfkm'nfO{ ;hfosf] efuL slQsf] 7fGg' eof] < -cfkm'n] s]xL ug{ g;Sg'df / s]xL s'/f lau|]sf]df_

slxNo} klg 7flgg============================================================ )

w/} 7fg] ======================================================================= !

;w}+ h;f] 7fg]================================================================== @

hLjgsf] x/]s s'/fdf 7fg]================================================== #

**BDI. 7** uPsf] b'O{ xKtfdf tkfO{nfO{ cfkm'b]lv slQsf] jfSs÷lbSs nfUof] <

slxNo} jfSs nfu]g ================================================================== )

clncln jfSs nfUof] ======================================================================= !

;w}+ jfSs nfUof] ====================================================================== @

cfkm'nfO{ g}+ 3[0ff nfUof]=============================================================== #

=

**BDI. 8** uPsf] b'O{ xKtfdf tkfO{n] cfkm\gf] ljutsf ulNtx? -k|lt slQsf] lhDd]jf/_ sltsf] dxz'; ug'{ eof]<

ulNt dxz'; ul/g =========================================================================== )

clncln ulNt dxz'; u/]=================================================================== !

w]/} ulNt dxz'; u/]====================================================================== @

;w} ulNt dxz'; u/]================================================================ #

**BDI. 9** uPsf] b'O{ xKtfdf tkfO{n] cfkm'nfO{ slQsf] xfgL k'Øfpg rfxg' eof] <

-h:t}M cfTd xTof ug{ dg nfUg], nfu'kbfy{ ;]jg ug]{_

cfkm'nfO{ s'g} klg xfgL k'Øfpg rflxg ===============================================)

xfgL k'Øfpg rfx]+ t/ s]xL ug{ ;lsg == =============================================!

d}n] cfkm'nfO{ dfg{ rfxGy]+==================================================================@

d}n] df}sf kfPsf] eP cfTdxTof uy{]=+===================================================#

**BDI. 10** uPsf] b'O{ xKtfdf tkfO{ slQsf] ?g' eof] <

k6Ss} /f]Og ===================================================================================== )

clncln /f]PF ========================================================================= !

; ;fgf] s'/fdf klg /f]PF===================================================================== @

c;fWo} ?g dg nfu]/ klg ?g ;lsg==================================================== #

**BDI. 11** uPsf] b'O{ xKtfdf tkfO{nfO{ slQsf] emsf]{ nfUof]<

slxNo} emsf]{ nfu]g=========================================================================== )

klxn] eGbf a9L emsf]{ nfUof]=============================================================== !

;w}+ emsf]{ nfu]sf] lyof]===================================================================== @

c;fWo} emsf]{ nfu]/ ;xg} ;lsg=========================================================== #

**BDI. 12** uPsf] b'O{ xKtfdf tkfO{nfO{ s'g} sfd ug{ dg gnfUg] slQsf] eof] . -h:t}M c?;Fu xfF;v]n ug{, sfd ug{_

slxNo} dg gnfUg] ePg=====================================================================)

clncln dg gnfUg] eof]= ==================================================================!

w]/} dg gnfUg] eof]============ ==== =========================================================@

;w} dg gnfUg] eof]===========================================================================#

**BDI. 13** uPsf] b'O{ xKtfdf tkfO{nfO{ lg0f{o lng slQsf] uf¥xf] eof] <

slxNo} uf¥xf] ePg ================================================== )

clncln uf¥xf] eof]================================================ !

w]/} uf¥xf] eof]========================================================== @

;w} uf¥xf] eof]================================================== #

**BDI. 14** uPsf] b'O{ xKtfdf tkfO{nfO{ cfkm' slQsf] Joy{÷g/fd|f] ePF h:tf] nfUof] <

slxNo} Joy{÷g/fd|f] eP h:tf] nfu]g ====================== )

clncln Joy{÷g/fd|f] eP h:tf] nfUof]====================== !

w]/} Joy{÷g/fd|f] e‘P h:tf] nfUof]================================= @

;w} Joy{÷g/fd|f] e‘P h:tf] nfUof ==================== =============#

**BDI.15** uPsf] b'O{ xKtfdf tkfO{n] slQsf] sfd ug{ ;Sg'eof] <

klxn] hlQs} sfd ug{ ;s]+ =============================================================== )

klxn] h:tf] sfd ug{ ;lsg ======= =================================================== !

sfd ug{ Psbd uf¥xf] eof] ============================================================== @

s]xL klg sfd ug{ ;lsg ================================================================ #

**BDI. 16** uPsf] b'O{ xKtfdf tkfO{ slQsf] ;'Tg' eof] <

klxn] hlQs} ;'t]+ ====== ==================================================================== )

klxn] h:tf] ;'Tg ;lsg====== ========================================================== !

aLrljrdf Ao'em]/ lgbfpg ;lsg =============================================== @

slQklg lgbfpg ;lsg============================================================== #

**BDI.17** uPsf] b'O{ xKtfdf tkfO{ slQsf] yfSg' eof] <

k6Ss} yflsg ====== ========================================================================= )

klxnf eGbf a9L yfs]+=========== ======================================================== !

h] ubf{ klg yfSby]+ ======================================= ================================= @

Psbd} yfs]/ s]lx klg ug{ ;lsg ====================================================== #

**BDI.18** uPsf] b'O{ xKtfdf tkfO{nfO{ vfg slQsf] ?rL eof] <

klxn] hlQs} ?rL eof] ======= ====== ===================================================== )

klxn] hlt ?rL ePg ====== == ============= =========================================== !

Psbd} sd ?rL eof] ======================================= =========================== @

k6Ss} ?rL ePg============================= =============================================== #

**BDI. 19** uPsf] b'O{ xKtfdf tkfO{sf] tf}n 36]sf] slQsf] dxz'; ug'{ eof]] <

36]sf] lyPg ========= ======= ====== ===========================================================)

clncln 36]sf] lyof] -% s]=hL=_==============================================================!

w]/} 36]sf] lyof] -!) s]=hL=_===================================================================@

Psbd} 36]sf] lyof] -!% s]=hL=_===== ========================================================#

**BDI. 20** uPsf] b'O{ xKtfdf tkfO{nfO{ cfkm\gf] :jf:Yosf] lrGtf slQsf] nfUof] <

k6Ss} lrGtf nfu]g =============================================================================)

clncln lrGtf nfUof]===========================================================================!

w]/} lrGtf eP/ c? s]lx ;f]Rg ;lsg ======================================================@

;w} lrGtf ePsf] sf/0fn] s]xL xf]z g}+ ePg =============================================#

**BDI. 21** uPsf] b'O{ xKtfdf tkfO{n] cfkm\gf] hLjg ;fYfL ->Ldfg÷>LdtL_ sf] af/]df slQsf] ;f]Rg' eof] <

klxn] hlQs} ;f]r]+ ============== ==============================================================)

klxn] eGbf sd ;f]r]===========================================================================!

Tolt jf:tf ePg ==================== =========================================================@

jf:tf g}+ ePg ========================== ========================================================#

**v08 #**

ca d s'g} JolQm lrGtfdf x'Fbf k|fo b]lvg] s]lx nIf0fx?sf] af/]df 5nkmn ug{ uO/x]sf] 5' . s[kof lt nIf0fx?n] tkfO{nfO{ klg uPsf] @ xKtfdf slQsf] lk/Nof] -b'Mv lbof]_ atfO{ lbg' xf]nf .

**BAI.1** uPsf] @ xKtfdf tkfO{nfO{ z/L/ emDemdfpg] slQsf] eof] <

x'Fb} ePg ==========================================================================================)

clncln eof]= ===================================================================================!

w]/} g} eof] t/ ;xg ;Sg]=====================================================================@

;xg} g;Sg] u/L eof] ==========================================================================#

**BAI.2** uPsf] @ xKtfdf tkfO{nfO{ z/L/ tftf] ePsf] dxz'z slQsf] eof] <

x'Fb} ePg ==========================================================================================)

clncln eof]= ===================================================================================!

w]/} g} eof] t/ ;xg ;Sg]=====================================================================@

;xg} g;Sg] u/L eof] ==========================================================================#

**BAI.3** uPsf] @ xKtfdf tkfO{nfO{ v'§f n/al/g] slQsf] eof] <

x'Fb} ePg ==========================================================================================)

clncln eof]= ===================================================================================!

w]/} g} eof] t/ ;xg ;Sg]=====================================================================@

;xg} g;Sg] u/L eof] ==========================================================================#

**BAI.4** uPsf] @ xKtfdf tkfO{nfO{ cf/fd ug{ slQsf] uf¥xf] eof] <

x'Fb} ePg ==========================================================================================)

clncln eof]= ===================================================================================!

w]/} g} eof] t/ ;xg ;Sg]=====================================================================@

;xg} g;Sg] u/L eof] ==========================================================================#

**BAI.5** uPsf] @ xKtfdf tkfO{nfO{ g/fd|f] 36gf 36\g] xf] sL eGg] dxz'z slQsf] eof] <

x'Fb} ePg ==========================================================================================)

clncln eof]= ===================================================================================!

w]/} g} eof] t/ ;xg ;Sg]=====================================================================@

;xg} g;Sg] u/L eof] ==========================================================================#

**BAI.6** uPsf] @ xKtfdf tkfO{nfO{ l/Ë6f nfUg] slQsf] eof] <

x'Fb} ePg ==========================================================================================)

clncln eof]= ===================================================================================!

w]/} g} eof] t/ ;xg ;Sg]=====================================================================@

;xg} g;Sg] u/L eof] ==========================================================================#

**BAI.7** uPsf] @ xKtfdf tkfO{nfO{ d'6' 9's9's ug{] slQsf] eof] <

x'Fb} ePg ==========================================================================================)

clncln eof]= ===================================================================================!

w]/} g} eof] t/ ;xg ;Sg]=====================================================================@

;xg} g;Sg] u/L eof] ==========================================================================#

**BAI.8** uPsf] @ xKtfdf tkfO{nfO{ cl:y/ x'g] -stfhf‘p÷s] u/f}+ s;f] u/f}+_ slQsf] eof] <

x'Fb} ePg ==========================================================================================)

clncln eof]= ===================================================================================!

w]/} g} eof] t/ ;xg ;Sg]=====================================================================@

;xg} g;Sg] u/L eof] ==========================================================================#

**BAI.9** uPsf] @ xKtfdf tkfO{nfO{ Psbd} em;Ë -tl;{g]_ x'g] slQsf] eof] <

x'Fb} ePg ==========================================================================================)

clncln eof]= ===================================================================================!

w]/} g} eof] t/ ;xg ;Sg]=====================================================================@

;xg} g;Sg] u/L eof] ==========================================================================#

**BAI.10** uPsf] @ xKtfdf tkfO{nfO{ To;} -ljgfsf/0f_ cflQg] slQsf] eof] <

x'Fb} ePg ==========================================================================================)

clncln eof]= ===================================================================================!

w]/} g} eof] t/ ;xg ;Sg]=====================================================================@

;xg} g;Sg] u/L eof] ==========================================================================#

**BAI.11** uPsf] @ xKtfdf tkfO{nfO{ 3fF6Ldf s]xL c8\lsPsf] h:tf] dxz'z x'g] slQsf] eof] <

x'Fb} ePg ==========================================================================================)

clncln eof]= ===================================================================================!

w]/} g} eof] t/ ;xg ;Sg]=====================================================================@

;xg} g;Sg] u/L eof] ==========================================================================#

**BAI.12** uPsf] @ xKtfdf tkfO{nfO{ xft sfDg]÷afpl8g] slQsf] eof] <

x'Fb} ePg ==========================================================================================)

clncln eof]= ===================================================================================!

w]/} g} eof] t/ ;xg ;Sg]=====================================================================@

;xg} g;Sg] u/L eof] ==========================================================================#

**BAI.13** uPsf] @ xKtfdf tkfO{nfO{ lhp sfDg] slQsf] eof] <

x'Fb} ePg ==========================================================================================)

clncln eof]=====================================================================================!

w]/} g} eof] t/ ;xg ;Sg]=====================================================================@

;xg} g;Sg] u/L eof] ==========================================================================#

**BAI.14** uPsf] @ xKtfdf tkfO{nfO{ cfkm\gf] xf]; -;Gt'ng_ x/fpnfsL eGg] dxz'z slQsf] eof] <

x'Fb} ePg ==========================================================================================)

clncln eof]= ===================================================================================!

w]/} g} eof] t/ ;xg ;Sg]=====================================================================@

;xg} g;Sg] u/L eof] ==========================================================================#

**BAI.15** uPsf] @ xKtfdf tkfO{nfO{ ;f; km]g{ uf¥xf] x'g] slQsf] eof] <

x'Fb} ePg ==========================================================================================)

clncln eof]= ===================================================================================!

w]/} g} eof] t/ ;xg ;Sg]=====================================================================@

;xg} g;Sg] u/L eof] ==========================================================================#

**BAI.16** uPsf] @ xKtfdf tkfO{nfO{ dl/Pnf sL eGg] 8/ slQsf] eof] <

slxn] ePg ======================================================================================)

clncln eof]= ===================================================================================!

w]/} g} eof] t/ ;xg ;Sg]=====================================================================@

;xg} g;Sg] u/L eof] =========================================================================#

**BAI.17** uPsf] @ xKtfdf tkfO{nfO{ 8/ nfUg] slQsf] eof] <

x'Fb} ePg ==========================================================================================)

clncln eof]= ===================================================================================!

w]/} g} eof] t/ ;xg ;Sg]=====================================================================@

;xg} g;Sg] u/L eof] ==========================================================================#

**BAI.18** uPsf] @ xKtfdf tkfO{nfO{ k]6 kf]Ng] ;d:of slQsf] eof] <

x'Fb} ePg ==========================================================================================)

clncln eof]= ===================================================================================!

w]/} g} eof] t/ ;xg ;Sg]=====================================================================@

;xg} g;Sg] u/L eof] ==========================================================================#

**BAI.19** uPsf] @ xKtfdf tkfO{nfO{ a]xf]z x'g] slQsf] eof] <

x'Fb} ePg ==========================================================================================)

clncln eof]= ===================================================================================!

w]/} g} eof] t/ ;xg ;Sg]=====================================================================@

;xg} g;Sg] u/L nuftf/ eof] ==============================================================#

**BAI.20** uPsf] @ xKtfdf tkfO{nfO{ l/; p7\bf of lrGtf nfUbf d'v /ftf] slQsf]] eof] <

x'Fb} ePg ==========================================================================================)

clncln eof]= ===================================================================================!

w]/} g} eof] t/ ;xg ;Sg]=====================================================================@

;xg} g;Sg] u/L eof] ==========================================================================#

**BAI.21** uPsf] @ xKtfdf tkfO{nfO{ udL{ geP klg kl;gf cfpg] slQsf] eof] <

x'Fb} ePg ==========================================================================================)

clncln eof]= ===================================================================================!

w]/} g} eof] t/ ;xg ;Sg]=====================================================================@

;xg} g;Sg] u/L eof] =========================================================================#
