# Supplementary material for: Anxiety and depression among people living in quarantine centers during COVID-19 pandemic: A mixed method study from western Nepal
Source: PLoS One. 2021 Jul 9;16(7):e0254126. doi: 10.1371/journal.pone.0254126 (PMC8270129; doi:10.1371/journal.pone.0254126)
Supplement: S4 Appendix — (DOCX) [file pone.0254126.s005.docx]

**Interview Guide for IDI for quarantined participants**

**IDI conduction date:**

**District:**

**Interviewee: Returnee** Migrants (quarantine)

**Name of moderator:**

**Interview start time:**

**Interview end time:**

**Place of interview:**

**Recorded: Yes**

| **SN** | **Questions/Variable** | **Response/Code** | **Skip** |
| --- | --- | --- | --- |
| A | Demographic Information |  |  |
|  | Address |  |  |
|  | District – |  |  |
|  | Palika |  |  |
|  | Ward # |  |  |
|  | Urban/Rural |  |  |
|  | Age |  |  |
|  | Sex |  |  |
|  | Caste/ethnicity |  |  |
|  | Religion |  |  |
|  | Education |  |  |
|  | Income |  |  |
|  | Marital status |  |  |
|  | No. of Family members |  |  |
|  | Duration of quarantine (in days) |  |  |
|  | Duration of work abroad(e.g. India) |  |  |

**Detail notes:**

1. Let’s start the interview. First of all, I will start the interview about life in general.

Prompts:

How did you arrive in this place (camp)?

Tell me about your life here?

How would you rate your life here? Very good/ good / okay / bad / very bad?

Are you coping?

2. Tell me about your accommodation while you are in quarantine (or in camp).

Prompts:

With how many people are you sharing room?

Who has arranged this place to live for you?

What facilities do you have in this place? For instance, TV, internet, toilet.

**3.** What changes have you experienced in your life after being quarantined? (before and after).

Prompts:

For example: changes in your life style, health, economic status etc.?

How do you manage your daily food (breakfast/lunch/meal etc)?

Who provides food?

How is the quality of food?

What is your financial status in quarantine?

Did you have good income working abroad?

4. Who is looking after people like you in quarantine?

Prompts:

How do they behave towards you?

Do health workers visit you (this place) to check your health status?

How do health workers treat you?

5. How are you spending your time here?

Prompts:

What do you mean?

Do you consume alcohol while you are in quarantine?

Do you smoke while you are in quarantine?

6. Now, let’s talk about epidemic diseases (e.g COVID-19)

Prompts:

Do you know about this disease?

How did you know about it?

Do you think this epidemics is risk for you and your family?

Why do you think it is risk for you and your family?

How it transfers or how people get it?

What are the transmission routes?

How it can be prevented?

7. Now, let’s talk about health.

Prompts:

Do you have any health problems?

What are general health problem among Nepalese migrants whilst in quarantine?

What are the major health problems of migrants while quarantine?

[For interviewer: you should ask subsequent question based on their answer.]

8. What do you know about stress or tension?

Prompts:

Tell me about your stress or tension here in quarantine (if any)?

Probe: problem like sleeping disturbances, irritation, anger while quarantine

How do you cope with such problem if you have any?

What kinds of stress or tension do other Nepali migrants experience because of quarantine?

9. Let’s talk about health services including mental health.

Prompts:

Do you receive any health care support in quarantine?

How friendly are health care workers?

Are you confident to speak with them?

What about quality of their services?

Do they provide counselling services for you?

What safety measures do you follow to avoid infection?

10. Let’s talk about access to healthcare in your community.

How easy is it for any Nepali in quarantine experiencing stress or tension to access healthcare?

Prompts:

Do know they the location of health center?

Do they have easy transport?

Can they afford money to reach hospitals?/can they afford money for treatment?

Can they share their problems with doctors/nurses?

Can they get quality of health services from health centers?

11. What would be the appropriate way to behave the people who are suffering from mental illness (who are in stress/tension) or problems?

12. Are there any mental health related services in your village/town or around?

13.What are the key challenges to get mental health related services in your community (in Nepal)?

Ending Interview!!!

Thank you very much for participating in this study.

**Interview Guide for IDI for quarantined participants in Nepali Language**

cGt{jftf{ lgb{]lzsf

!= cj s'/fsfgL ;'? u/f} x} . ;j{k|yd d clnslt tkfO{ cfkm\gf] jf/]df hfGg rfxG5' .

*tkfO{ s;/L, lsg of] 7fp+df cfOk'Ug eof]< oxf+ tkfO{ nfO{ s:tf] nflu /x]sf] 5 < tkfO{ o;sf] j;fO{ nfO{ s'g ?kdf lng' x'G5 < /fd|f], g/fd|f], jf l7s} < oxf+ j:g ;xh dxz'; ul//fVg' ePsf] 5 5}g <*

@= tkfO{ Sjf/]g6fOgdf j:bfsf] j;fO{ s:tf] eof] clnslt jtfO{lbg'; g .

*slt hgf ;+u sf]7fdf ;+u} j:g' kb{Yof] < oxf+ j:g s;n] jGbf]j:t ulb{of] < oxf+ s] s:tf ;'ljwfx? 5g\ < h:t} /]l8of], l6le, OG6/g]6, 6\jfO{n]6, ;/;kmfO{ cflb .*

#= tkfO{n] lhjgdf, lhjg z}nLdf, cfly{s cj:yfdf s]lx kl/j{tg kfpg'eof] hj tkfO{ Sjf/]g6fOgdf j:g' eof] <

*olx s;/L vfgf, kfgL vfhfsf] Joj:yf ul/Psf] 5 clnslt jtfO{lbg'; g . vfgf s;n] lbG5 < vfgfsf] u'0f:t/ s:tf] 5 < oxf+ j:bf oxf+sf] cfly{s cj:yf s:tf] 5 < tkfO{ ljb]zdf sfd ubf{ cfly{s cj:yf, cfDbfgL s:tf] lyof] <*

$= tkfO{ h:tf JolQx?nfO{ Sjf/]g6fOgdf s;/L Jojxf/ ul/Psf] h:tf] nfU5 <

*Jojxf/, :jf:Yo ;]jf k|jfx ubf{sf] s'/f, :jfYosdL{sf] Jojxf/*

%= tkfO{ oxf+ s;/L lbg ljtfO{ /fVg' ePsf] 5 <

*h:tf] /dfOnf] ug{], hf8/S;L vfgL, w'd|kfg ug{] cflb .*

^= clnslt sf]le8 !(sf] s'/f u/f} g .

*of] /f]u jf/]df s] yfxf 5 < s;/L yfxf kfpg' eof] < tkfO{ cfkm' jf cfkm\gf] kl/jf/nfO{ of] /f]usf] hf]lvd /x]sf]df s] 7fGg' x'G5 < of] s;/L ;b{5 h:tf] nfU5 < /f]syfd s] xf]nf <*

&= clnslt :jf:Yosf] jf/]df s'/fsflg u/f} g .

*tkfO{sf] :jf:Yodf s]lx ;d:ofx? 5g\ ls< oxf+ Sjf/]g6fOgdf j;]/ kf] of] ;d:of ePsf] h:tf] nfU5 ls <*

*= clnslt tgfj jf lrGtfsf] jf/]df s'/f u/f} g x} .

*Sjf/]g6fOgdf j:bf clnslt lrGtf jf tgfj ePsf] < h:tf] lgGb|df ;d:of ePsf], lemhf] nfu]sf], l/; p7]sf], cyjf oxf j:gsf] nflu ;d:of ePsf] jf cGo s'g} 5g ls < h:tf] oxf+ cfPsf c? dflg;x?n] s] s:tf ;d:ofx? ef]U5g h:tf] nfU5 <*

(= cj oxf+ pknJw ePsf :jf:Yo ;]jfx?sf] jf/]df clnslt s'/f u/f} g .

*oxf+ j:bf s'g} k|sf/sf] :jf:Yo ;]jfx? kfpg' ePsf] < :Jff:yosdL{n] tkfO{nfO{ clnslt /fd|f] Jojxf/ u/]sf] . tkfO{ nfO{ c?;+u jf]Ng s]lx ;+sf]r nfu]sf] . plgx?n] lbg] ;]jfsf] jf/]df s] eGg' x'G5 < plgx?n] s'g} k|sf/sf] k/fdz{ lbP ls < ;+s|d0fjf6 jRg tkfO{n] s] s:tf ;jfwfgLx? ckgfpg' eof] <*

!)= clnslt tkfO{ ;d'bfodf jf 3/ kmls{;s]kl5 sf] cj:yf jf/]df s'/f u/f} g .

*:jf:Yo ;]jf k|fKt ug{ sltsf] ;xh 5 < :jf:yo ;]jf lbg] ;+:yf;Dd k'Ug slQsf] ;xh 5< cyjf Sjf/]g6fOgdf j:bf sltsf] :jf:Yo ;+:yf ;Dd k'Ug ;xh lyof] < s] tkfO{n] cfkm\gf] ;d:ofx? :jf:YosdL{x? ;+u ;xh} /fVg ;Sg' eof] < plgx?n] lbPsf] ;]jfsf] u'0f:t/ s:tf] 5 h:tf] nfU5 <*

!!= s;/L o:tf lrGtf jf tgfjjf6 u'lh|Psf dflg;x?nfO{ Jojxf/ ul/g'kb{5 h:tf] nfU5 <

!@= clnslt tkfO{sf] ufp+ zx/df o:tf] ;]jf lbg] ;+:yfx? 5g ls < tkfO{nfO{ cfkm\gf] ufp+ zx/df dfgl;s :jf:Yo ;DjlGw s]lx k/fdz{ rflxPsf] 5 eg] s;/L ;xof]u ug{ ;lsG5 h:tf] nfUb5 <

!#= cj cGTodf d}n] tkfO{nfO{ w]/} s'/f ;f]WP . tkfO{nfO{ d;+u s]lx ;f]Wg jf hfGg dg nfu]sf] 5 <

**wGojfb ...**
