# Supplementary material for: Anxiety and depression among people living in quarantine centers during COVID-19 pandemic: A mixed method study from western Nepal
Source: PLoS One. 2021 Jul 9;16(7):e0254126. doi: 10.1371/journal.pone.0254126 (PMC8270129; doi:10.1371/journal.pone.0254126)
Supplement: S5 Appendix — (DOCX) [file pone.0254126.s006.docx]

Interview Guide for IDI for Health Workers

IDI conduction date:

District:

Interviewee: Health care workers providing services in in quarantine centers

Name of Interviewer:

Interview start time:

Interview end time:

Place of interview:

Recorded: Yes

Socio-demographic Information

| SN | Questions/Variable | Response/Code | Remarks |
| --- | --- | --- | --- |
| A | Demographic Information |  |  |
|  | Address |  |  |
|  | District – |  |  |
|  | Palika |  |  |
|  | Ward # |  |  |
|  | Urban/Rural |  |  |
|  | Age |  |  |
|  | Sex |  |  |
|  | Caste/ethnicity |  |  |
|  | Religion |  |  |
|  | Education |  |  |
|  | Income |  |  |
|  | Marital status |  |  |
|  | No. of Family members |  |  |
|  | Duration of working in quarantine center(in days) |  |  |

Detail notes:

1 Explain the study and ask for the consent, then record the consent when you turn on the microphone.

*Eg: “Thank you for talking to me today… are you happy to take part in the study?”*

**Turn the recorder on**

*Ok, so I have turned the microphone on. I just want to ask you again, are you happy to take part in this study by talking with me today?*

#### 2. Let’s start the interview. How many people do you approximately provide service to in the quarantine centers every day?

#### Prompts: What are the common health problems, how many people seek health services (preventive and curative)

#### 3. How have you been looking after people those being quarantined?

#### Prompts: Services you provide, health checkups and treatments, counselling, referrals

#### 4. How do the quarantined people behave towards you?

#### Prompts: Their comfort in approaching you when needed, do they express satisfaction/dissatisfaction to the health and other services provided?

#### 5. Would you please tell me your thoughts on the life of quarantined people and their stay here?

#### Prompts: their accommodation, sanitation, and hygiene, sharing room, living place, facilities, entertainment, social interactions, internet facilities

#### 6. What is your observation on quarantined peoples’ awareness about Covid-19?

#### Prompts: About this disease, mode of transmission, risks of getting infected and dying, preventive measures, source of information.

####

#### 7. Have you observed mental health challenges in those people while in quarantine?

#### Prompts: Stress or tension here in quarantine (if any)? What are their main stressors? Problem like sleeping disturbances, irritation, anger while quarantine? How do they cope with such problem if they have any? What kinds of stress or tension do Nepali migrants experience because of quarantine?

#### 8. What do you think about the quality of health services provided to the quarantined people?

#### Prompts: Quality/Availability of routine health services and mental health services? Counselling services? Safety measures to followed to avoid infection?

#### 9. Do you have any suggestion to strengthen the routine and mental health services to the quarantined population?

#### Thank you very much for participating in this study.

#### Ending Interview!!!

**Interview Guide for Health Workers- IDI in Nepali Language**

cGt{jftf{ lnPsf] ldlt M

lhNnf M

cGt{jftf{ lnOPsf] JolQ M Sjf/]g6fOgdf sfd ug{] :Jf:YosdL{

cGt{jftf{ lng]sf] gfd M

cGt{jftf{ ;'? ePsf] ;do M

cGt{jftf{ ;lsPsf] ;do M

cGt{jftf{ lnOPsf] :yfg M

cGt{jftf{ /]s8{ ul/Psf] M 5

**JolQut tyf hg;+VosL ;DjlGw ljj/0f**

| **l;=g** | **k\|Zgx?** | **hfjfkm** | **s}lkmot** |
| --- | --- | --- | --- |
| s | hg;+VosL ;DjlGw ljj/0f |  |  |
|  | 7]ufgf |  |  |
|  | lhNnf |  |  |
|  | kflnsf |  |  |
|  | j8f g+ |  |  |
|  | ufp+ jf zx/df j;f]jf; ug{] |  |  |
|  | pd]/ |  |  |
|  | ln+u |  |  |
|  | hft jf hfthftL |  |  |
|  | wd{ |  |  |
|  | lzIff |  |  |
|  | dfl;s cfDbfgL |  |  |
|  | j}jflxs cj:yf |  |  |
|  | kl/jf/ ;b:osf] ;+Vof |  |  |
|  | Sjf/]g6fOgdf sfd u/]sf] hDdf cjlw |  |  |

gf]6 M =========================================================================================================================

=========================================================================================================================

=========================================================================================================================

=========================================================================================================================

cGt{jftf{ lgb{]lzsf

!= **o; cWoogsf] jf/]df k|Zg ub}{, ;xefuLsf] ;xdltsf] nflu ;f]Wg'xf];\ . To;kl5 ;xefuLnfO{ dfOqm|f]kmf]gdf plgx?sf] cGt{jftf{ /]s8{ ug{ nfluPsf] hfgsf/L lbg'xf];\ / o;sf] nflu ;xdlt dfUg'xf];\ .**

h:t} M cfh d;+u s'/fsfgL ug{ ;do lbg'ePsf]df wGojfb . tkfO{ o; cWoogdf ;xefuL x'g kfpbf+ v';L x'g'x'G5 lg <

**/]s8{/ cg ug{'xf];\ .**

x'G5, cj d}n] oxf+sf] cGt{jftf{ ;'? u/]+ . d km]/L klg cfh tkfO{ o; cWwogdf ;xefuL x'g OR5's x'g'x'G5 eGg] hfGg rfxG5' <

**@=ca s'/f ;'? u/f} x}, oxf+ j:g] dflg;x?sf] nflu cGbfhL slt dflg;x?n] sf dub{5g\ <**

*oxf+sf] dflg;x?sf] d'Vo :jf:Yo ;d:ofx?, slt dflg;x?n] s;/L :jf:Yo ;d:ofx? kfO{/fv]sf 5g\ <*

**#= tkfO{sf] Sjf/]g6fOgdf j:g] dflg;x?sf] ;]jf cGt{ut s] s:tf lhDj]jf/Lx? kb{5g\ <**

*;]jfx? h'g oxfn] lbg'x'G5, :jf:Yosf] hf+r tyf pkrf/, ;/;Nnfx tyf k|]if0fsf s'/fx?*

**$= Sjf/]g6fOgdf j:g] dflg;x?n] s;/L oxf+ ;+u Jojxf/ ub{5g\ <**

**pxf+x?nfO{ cfjZos kbf{ oxf+ ;+u s'/f ug{ sltsf] ;xh dxz'; ub{5g\ < plgx?sf] :jf:yo tyf cGo ;]jf k|jfx ubf{ s] s:tf ;Gt'i7L tyf c;Gt'i7Lx? 5g <**

**%= oxfFsf] j;fO{ tyf a:gsf] nflu cfjf;sf] Aojf:yf tkfO{ nufPt oxfF a:g] cGo JolQmx?nfO{ s:tf] /x]sf] 5 <**

vfg] j:g] 7fp+, ;/;kmfO{, *sf]7f ldlnh'nL k|of]u ug{'kg]{, a:g] 7fpFsf] ;'ljbf, dgf]/Ghg , ;fdflhs e]63f6, OG6/g]6 nufPt cGo ;'ljbfx?*

**^=tkfOnfO{ oxfF ePsf JolQmx?df sf]le8 ;DalGw slQsf] hfgsf/L 5 h:tf] nfU5 <**

*s:tf] /f]u xf] < pxfx?n] of] /f]usf] af/]df s;l/ hfgsf/L kfpg'eof] < of] /f]un] pxfx? nufPt pxfx?sf] kl/jf/df s] s:tf] hf]lvd lgDTofp5< /f]u ;g]{ dfWod / o;sf] /f]syfd s] xf]<*

**&= oxf+ j:g] dflg;x?sf] dfgl;s :jf:Yo ;d:of tyf :jf:Yosf ;d:ofx?df s] s:tf r'g}ltx? /x]sf 5g\ <**

*oxfF s'g} tgfa tyf lrGtf ln/fVg] ,lgG›f gkg]{, lemhf] nfUg], l/if pl7/xg] h:tf] ;d:of ePsf] sf]lx e]l6Psf] 5 ls < tkfO{x? To:tf ;d:ofx? s;/L ;dfwfg ug{'x'G5 / ul//xg' ePsf] 5 < sf]le8 eP/ oxfF a:gfn] g]kfn lelqPsf JolQmx?df s:tf] vfnsf] tgfa l;h{gf ePsf] 5<*

***=ca Ps}l5g oxfF pknAw x'g] dfgl;s :jf:Yo nufPt cGo :jf:Yo ;]jfx?sf] af/]df s'/f u/f}**

*oxfF s] s:tf] k|fylds :jf:Yo ;]jfx? pknAw 5g\ < d}qLk"0f{ :jf:YosdL{x? x'g'x'G5 ls x'g'x'Gg < uf]klgotf sfod s:tf] 5< :jf:Yo ;]jf sf] u'0f:t/ s:tf] 5 < k/fdz{sf] ;]jf / Jofofdsf] Joj:yf, OG6/g]6sf] ;'lawf nufPt cGo dgf]/+hgsf] ;'lawf <*

**(=tkfOsf] ljrf/df dfgl;s la/fdLx?nfO{ s:tf] Jojxf/ ckgfpg' pko'Qm x'G5 h:tf] nfU5 <**

*oxfn] o; cWoogdf ;xefuL x'g' ePsf]df wGojfb .*

**;dfKt**
